# Feasibility, experiences and outcomes of using DIALOG+ in primary care to improve quality of life and mental distress of patients with chronic conditions: an exploratory non-controlled trial in Bosnia and Herzegovina, Colombia and Uganda

**DOI:** 10.1186/s40814-021-00914-z

**Published:** 2021-09-30

**Authors:** Francois van Loggerenberg, Michael McGrath, Dickens Akena, Harriet Birabwa-Oketcho, Camilo Andrés Cabarique Méndez, Carlos Gómez-Restrepo, Alma Džubur Kulenoviĉ, Maja Muhić, Nelson K. Sewankambo, Hana Sikira, Stefan Priebe

**Affiliations:** 1 Unit for Social and Community Psychiatry, Queen Mary University of London, London, UK; 2 Department of Psychiatry, Makerere University College of Health Sciences, Kampala, Uganda; 3 Butabika National Referral Mental Hospital, Kampala, Uganda; 4 Department of Clinical Epidemiology and Biostatistics, Pontificia Universidad Javeriana, Bogotá, Colombia; 5 Department of Psychiatry and Mental Health, Pontificia Universidad Javeriana, Bogotá, Colombia; 6 Hospital Universitario San Ignacio, Bogotá, Colombia; 7 Clinical Center University of Sarajevo, Sarajevo, Bosnia and Herzegovina; 8 Department of Internal Medicine, Makerere University College of Health Sciences, Kampala, Uganda

**Keywords:** Global mental health, Primary care, Psychosocial interventions, Resource-oriented approach, LMICs, Solution-focused, Quality of life, DIALOG+

## Abstract

**Background:**

DIALOG+ is a resource-oriented and evidence-based intervention to improve quality of life and reduce mental distress. While it has been extensively studied in mental health care, there is little evidence for how to use it in primary care settings for patients with chronic physical conditions. Considering that DIALOG+ is used in existing routine patient-clinician meetings and is very low cost, it may have the potential to help large numbers of patients with chronic physical conditions, mental distress and poor quality of life who are treated in primary care. This is particularly relevant in low- and middle-income countries (LMICs) where resources for specialised services for such patients are scarce or non-existent.

**Methods:**

An exploratory non-controlled trial will be conducted to primarily assess the feasibility and acceptability and, secondarily, outcomes of delivering DIALOG+ to patients with chronic physical conditions and poor quality of life in primary care settings in Bosnia and Herzegovina, Colombia and Uganda. Thirty patients in each country will receive DIALOG+ up to three times in monthly meetings over a 3-month period. Feasibility will be assessed by determining the extent to which the intervention is implemented as planned. Experiences will be captured in interviews and focus groups with care providers and participants to understand acceptability. Quality of life, symptoms of anxiety and depression, objective social situation and health status will be assessed at baseline and again after the three-session intervention.

**Discussion:**

This study will inform our understanding of the extent to which DIALOG+ may be used in the routine care of patients with chronic physical conditions in different primary care settings. The findings of this exploratory trial can inform the design of future full randomised controlled trials of DIALOG+ in primary care settings in LMICs.

**Trial registration:**

All studies were registered prospectively (on 02/12/2020 for Uganda and Bosnia and Herzegovina, and 01/12/2020 for Colombia) within the ISRCTN Registry. ISRCTN17003451 (Bosnia and Herzegovina), ISRCTN14018729 (Colombia) and ISRCTN50335796 (Uganda). Protocol version and date: v2.0; 28/07/2020 (Bosnia and Herzegovina), v0.3 02/08/2020 (Colombia) and v1.0, 05/11/2020 (Uganda).

## Background

Chronic physical diseases, such as diabetes, cardiovascular disease, HIV/AIDS, and cancer are associated with increased psychological distress and reduced quality of life [1–4]. Chronic mental illnesses are often co-morbid with physical illnesses [5–10] due to shared risk factors, poorer treatment adherence, and bidirectional causality [11]. Identifying and treating mental distress in chronically ill patients has the potential to improve quality of life and functioning, despite other comorbidities [12]. This calls for the integration of mental and physical care to address the unmet mental health care needs for patients with chronic conditions.

Integrating mental and physical healthcare within primary care has again been highlighted as a priority [13] and some efforts are underway [14]. However, there are persistent challenges such as inadequate financing, a lack of skilled personnel, high patient load, and limited evidence surrounding the design and delivery of interventions that support improved mental health within the health systems of low- and middle-income countries (LMICs) [15–19]. In Africa, for example, generally, less than 1% of health budgets are spent on mental health [20], and integration of effective interventions in primary care could significantly improve access, provide patient-centred care, avoid fragmentation in the health services and potentially lessen stigma, whilst optimising both mental and physical health outcomes [21].

### DIALOG+ intervention

DIALOG+ is a resource-oriented and evidence-based intervention, which makes use of existing personal and social resources to improve the quality of life of patients with psychological distress by structuring the discussion that takes place during routine clinical meetings. It encourages self-reflection and expression and empowers patients to take actions to improve their mental health and social situation [22]. DIALOG+ was developed based on the quality of life research, concepts of patient-centred communication, developments in information technology, and components of solution-focused therapy to make routine meetings between patients with mental disorders and healthcare professionals therapeutically effective (more information at https://dialog.elft.nhs.uk) [22, 23]. Through a process of patient-centred assessment, clinicians ask patients to rate their satisfaction with eight life domains and three treatment aspects, on a seven-point scale ranging from ‘totally dissatisfied’ to ‘totally satisfied’. This is followed by a four-step, solution-focused approach that guides the clinician to explore the concerns raised by the patient and possible actions that can be taken by the patient and others to address these concerns. Specifically, the four steps outlined in the manual [24] are (1) *Understanding*, intended to allow the clinician and the patient to understand the patient’s situation by assessing why the rating is the one given and, for example, not lower, and discussing what is working; (2) *Looking forward*, where the patient is encouraged to imagine what changes (‘best case scenario’ and ‘smallest improvement’) they would like to see to their current situation; (3) *Exploring options*, to see what practical actions might lead to the desired change, this includes looking at things that the patient, the clinician and others can do; and (4) *Agreeing on actions*, where the clinician and patient agree on specific actions that are documented for review at the next session.

DIALOG+ is supported by an app available for a tablet or smartphone and provides assessment, planning, therapeutic intervention and evaluation in one procedure. The fact that the app is hosted on a tablet means that the patient is more actively involved in the meeting, and the tablet can be passed between the patient and the provider to support discussion. The scores are summarised and can easily be compared on the screen, and, if this is a subsequent visit, be compared to scores provided at earlier visits. This gives a rapid overview of the current problems and strengths in the patient’s life. As part of the intervention, providers are encouraged to provide positive feedback domains that are scored high or, at subsequent visits, any domains that show improvement. DIALOG+ requires minimal training and can be delivered by a broad range of professionals at all levels of the healthcare system.

In randomised controlled trials in Western Europe, DIALOG+ has been shown to significantly improve patient outcomes whilst reducing care costs [22, 23, 25]. However, whilst DIALOG+ has been extensively studied in mental health care, there is little evidence for how to use it in primary care settings for patients with chronic conditions. As DIALOG+ is a low-cost intervention, which mobilises existing personal and social resources, it may also be used to support effective community mental health care within a primary care setting. It could be used with patients with chronic physical disorders, particularly if these disorders and associated social problems impact patients’ quality of life. Given the flexibility of DIALOG+ and the short training required, it may be adapted and used for patients with chronic physical illness and poor quality of life in primary care settings. Applying an effective evidence-based and resource-oriented intervention such as DIALOG+ has the potential to improve outcomes for individuals with chronic conditions and make efficient use of limited resources in primary healthcare in LMICs. Within this setting, DIALOG+ may help to develop service capacity and improve clinical and social outcomes. This study will inform our understanding of the extent to which DIALOG+ may be used in the routine care of patients with chronic physical conditions in different primary care settings in LMICs.

### Aims and objectives

The primary aim of the exploratory study is to assess the feasibility and acceptability of using DIALOG+ for patients with chronic physical illness and poor quality of life in primary care settings in three LMICs. A secondary aim is to assess changes in key outcomes.

## Methods

### Design

This work developed from The NIHR Global Health Research Group on Developing Psycho-social Interventions in Low- and Middle-Income Countries (GLOBE), a multi-disciplinary collaboration between Queen Mary University of London, East London NHS Foundation Trust, and partners in Bosnia and Herzegovina (Clinical Centre, University of Sarajevo), Colombia (Javeriana University) and Uganda (Makerere University, College of Health Sciences) [26]. As part of this collaboration, GLOBE specifically set out to test resource-orientated approaches for addressing mental health globally. Central to the work completed by GLOBE were three randomised controlled trials of DIALOG+. Following the successful recruitment and implementation of DIALOG+ within services for people with serious mental illness, potential new and extension projects were discussed. Positive clinician and patient feedback and preliminary quantitative findings suggested the intervention improved quality of life, reduced psychiatric symptoms and increased empowerment. The team identified the needs of individuals with co-morbid long-term physical and mental health conditions, often treated within primary care, as an urgent and common priority in all three countries.

DIALOG+ will be applied in primary care settings in an exploratory non-controlled trial. The evaluation will use mixed methods, with outcomes measured at baseline and the end of the intervention period. Implementation processes will be informed by a stakeholder consultation based on actual experience of implementing the intervention in the previous DIALOG+ GLOBE trials [26]. The results will provide insight into refining the manualised training process and implementing the intervention across healthcare settings. The first stages of the study may involve minor modifications to DIALOG+ to ensure that it is useable in the primary care context in the participating LMICs. Potential adaptations may include suggested modifications to item wording and training material, especially around when and who delivers each component of the intervention.

A core research protocol was developed to ensure comparable implementation across countries and core outcomes, but there was some flexibility to adapt the protocol to be suitable for local implementation. Therefore, there are some small differences with regard to some of the measures and inclusion criteria. Additionally, the local teams identified primary care clinic populations that they were able to integrate the DIALOG+ work with. This is reported here with reference to the Spirit Guidelines [27] and with consideration of guidelines on the reporting of pilot and feasibility studies [28].

### Patients and procedures

We will target chronic non-communicable disorders such as diabetes, cardiovascular disease, chronic obstructive pulmonary disease and hypertension, as these conditions negatively impact on quality of life and are highly comorbid with mental distress [29]. Adult patients (18–65 years in Bosnia and Herzegovina and Colombia, 18+ in Uganda where the selected clinic population (diabetes and hypertension) was anticipated to be older) with at least one chronic condition and poor quality of life (< 5 on the Manchester Short Assessment of Quality of Life [MANSA], < 5.5 in Colombia where in our previous work, participants with mental illness tend to score slightly higher on quality of life). Additionally, participants must exhibit the capacity to provide informed consent, speak the local language, live within a 20-km radius of the clinic and have been attending the primary care clinic for at least 6 months.

Participants who do not meet these criteria or who are unwilling or unable to provide informed consent will not be eligible for participation.

For clinicians, they must be qualified health professionals currently working in the relevant primary care facilities with no plans to leave their post within 4 months.

### Consent

Individuals who respond to the study information with interest will be contacted and invited by phone or letter to attend a face-to-face meeting with a researcher. Researchers will discuss the study with interested individuals and answer any questions or concerns that are raised. At this stage, contact details will be confirmed, and availability ascertained for attendance of intervention sessions, interviews or appointments.

Prior to any data collection, all participants, including clinicians, will be asked to provide informed consent by signing and dating an informed consent form. Two copies of the written consent form will be signed by the participant and a member of the research team in order to proceed with study participation. The participant will keep one copy of the informed consent form and the research team will keep the other, storing it in a locked filing cabinet.

Specific procedures will be implemented in each country and reviewed and approved by the local ethics committee to determine how to facilitate and document consent for all participants, including those with low literacy.

All researchers will receive Good Clinical Practice training by members of the UK-based research team, or senior members of the local research team, or through online/official courses. The researchers will assess each patient's level of understanding during the recruitment and consent process, alongside discussion with the patient’s clinician where necessary. If any doubts about their capacity emerge during the recruitment process, or capacity to consent appears to change during their participation in the study, their capacity to consent will be re-evaluated before continuing with study participation.

### Sample size

We plan for a sample size of at least 30 participants enrolled per country. Central limit theory suggests 30 as a minimum sample size to enable meaningful parameter estimates in an exploratory study [30]. We anticipate that each of around five clinicians enrolled in each country will deliver the intervention to approximately six participants.

### Time periods

Once enrolled, patients will receive DIALOG+ at their routine primary care appointments, about once per month. Sessions will be delivered by the healthcare worker supported by an app on a tablet computer. The intervention period will be approximately three months, during which patients will attend up to three DIALOG+ sessions.

Data collection with all participants will take place at baseline and following the three-session intervention period. At baseline, the researchers will collect socio-demographic information from all participants, which, for patient participants, will include clinical characteristics.

At the conclusion of the intervention period, all clinicians and a subset of patients will be invited to attend an interview. The remaining patients will be asked to take part in focus group discussions. These discussions will follow a structured guide, and be conducted by trained and experienced researchers. Specific consent will be obtained to audio-record interviews and focus group discussions, which will be transcribed verbatim for analysis. These data will be used to capture the experiences of receiving or delivering the intervention in primary care and be used to assess the acceptability of the DIALOG+ in this context.

In addition to the research data collection, the research and clinical support staff will keep a record of their intervention sessions. These reports will include the date, duration of interventions and the topic of discussion. This information is also captured within the DIALOG+ app and can be retrieved by the research staff. The aim is to audio record at least one session per patient, which will be scored for adherence to the intervention, using the DIALOG+ Adherence Scale. Specific consent will be sought for these recordings. These data will be used to assess the feasibility of implementing the intervention in this context.

### Withdrawal

During the consent process, researchers will ensure that participants are aware of their right to decline participation at any stage of the research and that withdrawing participation will not affect their treatment or rights. Participants who withdraw will be able to ask for their data to be removed from the study, providing this occurs within the intervention period.

If patients withdraw from the intervention, then the research team will ask permission to contact them to take part in the follow-up assessment and post-intervention qualitative interview in order to capture valuable information regarding reasons for withdrawal, which might be associated with the intervention. This will be explained to patients during the consent process. The research team will respect the decision of patients who do not wish to be contacted for the follow-up assessment and post-intervention interview.

If a participant wishes to withdraw from the study, researchers will record the date of withdrawal and reason(s) for withdrawal if provided.

## Measures

### Capacity to consent at screening:

In Uganda, all participants will be assessed with the University of California, San Diego Brief Assessment of Capacity to Consent (UBACC); used to identify subjects with questionable capacity to consent to the specific research project. It has good internal consistency, inter-rater reliability, concurrent validity, high sensitivity, and acceptable specificity. It typically takes less than five minutes to administer and is easy to use and reliably score [31]. Participants will need to reach an adequate standard within three administrations of this instrument.

In Colombia and Bosnia and Herzegovina, researchers will implement an adapted capacity to consent checklist, based on that published by the British Psychological Society, for those participants who it is felt may lack the capacity to consent [32].

In all countries, responses to these measures will be used to structure discussions with participants to attempt to allow them to reach a standard required for enrolment.

### Recruitment and retention rates:

Process measures will be used to assess the feasibility of the intervention in this context. We will assess patient and clinician recruitment and attrition (reasons for refusal and loss to follow-up), frequency and duration of sessions. Feasibility criteria include at least 75% recruitment of the anticipated participants, coupled with at least 75% retention [28]. An additional feasibility criterion will be an average of at least two out of the three planned sessions completed.

### Key outcome measures

For all patients and clinicians: socio-demographic information will be collected at baseline.

The impact of the intervention on key outcomes will be evaluated by assessing changes in the following measures, assessed at baseline and at the end of the intervention period:

1. Quality of life will be measured using the Manchester Short Assessment of Quality of life (MANSA), a 16-item scale which assesses quality of life across different life domains [33].
2. Patient Health Questionnaire (PHQ-9 in Uganda and Bosnia and Herzegovina, PHQ-8 in Colombia (where the research team has more experience with this version of the PHQ and the scores are comparable [34]) is a brief depression scale with good sensitivity and specificity, and consists of the criteria upon which the diagnosis of DSM-IV depressive disorders is based. The scale will be scored as the PHQ-8 for the pooled analysis across countries.
3. Generalised Anxiety Disorder Assessment (GAD-7); has been used in primary care and has good validity, and reliability [35].
4. The Objective Social Outcomes Index (SIX) summarises objective indicators of social outcomes in mental health and has been shown to capture changes over time [36].
5. Physical Health Symptoms was designed for use in clinical practice and research, health policy evaluations, and general population surveys [37, 38] (the SF-36 will be used in Uganda and Bosnia and Herzegovina, and the SF-12 in Colombia which has been validated for use in Latin America [39, 40] and is shorter to administer).

### Participant experience

To assess acceptability, qualitative interviews and focus groups will be conducted to capture patient and clinician experiences of receiving and delivering the intervention, suggested adaptations to training material and supervision, barriers and facilitators of attendance and delivery, and the impact of the intervention on routine meetings.

## Settings

This study will be conducted in primary care settings in each country. These are arranged and managed differently in each country, and so will be described briefly here.

### Bosnia and Herzegovina

Health care is organised differently in the two entities of Bosnia and Herzegovina (BiH) – Federation of Bosnia and Herzegovina and Republic of Srpska. In the Federation of Bosnia and Herzegovina, where our study will take place, organisation of health care is delivered through the ten cantons with ten respective cantonal health ministries and a Federal ministry of health which has a guidance role [41, 42]. Continuity of care is a key characteristic of primary health care.

The Public Institution Health Centre of Sarajevo Canton is the largest institution in BiH providing primary health care [43]. The institution has nine community health centres, one in each municipality. Primary health care services involve family medicine and primary mental health care in the Community Mental Health Centres. Some secondary health care services are also available in this institution – internal medicine, neurology, ophthalmology, ear nose and throat, and dental medicine. The aim of primary health care is to make health services accessible and available for all the citizens, so the outpatient clinics are placed in the local communities.

Although Bosnia and Herzegovina is one of the most vulnerable societies in the region, it has made significant progress in the area of mental health care reform, which was launched in 1996 focusing on community-based mental health. BiH is the only country in South-East Europe that has set up a network of 74 community-based mental health centres which provide services to 3.8 million residents [44]. The centres employ multi-disciplinary teams comprising psychiatrists, psychologists, social workers and medical nurses; some centres, however, also employ occupational therapists, defectologists, speech therapists, somatotherapists, and child psychiatrists. These processes aim to build an effective, efficient and high-quality mental health service focused on the user needs, accessible to as many people as possible in the context of the integrated system of service delivery. The mental health care system needs to protect human rights, ensure gender equality and efficiently respond to the diverse needs of the population, especially of the most vulnerable groups.

This study will recruit patients from the Public Institution Health Care Centre of Sarajevo. Clinicians who will deliver the interventions are physicians who work at the primary health care centre of Sarajevo. The intervention, patient assessments, interviews and physician training will take place in the offices of primary health care centres which provide privacy and confidentiality.

### Colombia

In Colombia, the new model of care emphasises the role of primary care in the national public health strategy. The policy ‘PAIS’ (Plan de Atención Integral en Salud, or comprehensive health care policy) appears in this new model, which seeks to provide an integrated model of healthcare, in such a way that the entire territory has timely, effective and high-quality access, based on a health promotion and prevention strategy. This model is called MIAS (Modelo Integral de Atención en Salud, or comprehensive health care model) and proposes an evaluation of the health needs of a given territory (city, municipality, state) in such a way that, with the health insurance entities either EPS (Entidad Promotora de Salud, or Health Promoting Entity) or IPS (Institución Prestadora de Servicios, or Service Provider Institution), strategies are developed that address these needs. For this, the model proposes the development of comprehensive health care routes (RIAS, Red Integral de Atención en Salud). The first access route is provided by primary health care, which seeks to guarantee timely access to medical care. However, another function is to establish the specific care route by risk groups, to guarantee appropriate care. Within this, there are 16 risk groups, among which are those with mental health problems, violence-related issues and problems related to drug abuse. However, the capability of the primary care level to deal with chronic conditions has been limited. Increasing the capacity of primary care services to manage chronic conditions in an integrated way is a priority for the system. Comorbidity is managed in primary care as there is a lack of specialists within the healthcare system [45].

In Colombia, the study will be conducted in Bogotá and the clinicians and the patients will be recruited from Javesalud Institución Prestadora de Servicios (IPS, Service Provider Institution). The intervention and assessments sessions and interviews will take place in private rooms within clinics or other participating institutions. However, with COVID-19 restrictions in place (see section later) most sessions with patients are likely to be virtual (an attempt will be made to have the first DIALOG+ contact in person if possible), with care providers at the clinic providing most contacts by video-call or telephone.

### Uganda

Primary health care in Uganda is provided by both the public and private sectors with the public sector contributing approximately 60% [46]. The public sector has a referral system with the national referral hospitals as the final points of health services; however, this is often exploited due to a lack of a clear gatekeeper role [47]. Primary health care should be provided by the units in the Health Sub-district but this is not the norm [46]. Communities use the nearest health facility as their primary health care service and so those living near the referral hospitals use them as their primary care services without following the intended referral pathway [47]. In Uganda, this study will be carried out at the medical outpatient clinics of Masaka regional referral hospital and Mityana district hospital. The outpatient clinics are for chronic illnesses like diabetes and hypertension and are run by health workers that include doctors, clinical officers and senior nurses.

### SARS-CoV-2 (COVID-19) modifications

The COVID-19 pandemic has affected the three involved countries quite differently, although restrictions and social distancing have impacted all preparatory and planned activities. In the main studies [26], some follow-up and intervention sessions could not take place in person due to pandemic restrictions and social distancing. We have learned from these experiences, and the team is prepared to provide remote or socially distanced DIALOG+ and assessment visits as necessitated by in-country requirements. Initial plans are for most interactions to be conducted remotely in Colombia. The first DIALOG+ session will be delivered to patients face-to-face, if possible, whilst recruitment and follow up visits and assessments will be conducted via telephone or video conferencing. In Uganda and Bosnia and Herzegovina face-to-face contact is still possible and visits are subject to social distancing and infection control procedures, although the situation in Bosnia and Herzegovina is more uncertain due to increasing case rates. All sites have protocols and plans in place to implement appropriate social distancing or remote delivery to respond to local requirements and regulations which may change at any time. We anticipate this will allow us to refine procedures implemented in the initial studies, whilst also being able to reflect on whether remote delivery of DIALOG+ is feasible and acceptable and what impact this may have on the outcomes. All modifications to DIALOG+ delivery and assessment visits are carefully documented for reporting and analysis purposes. Modifications to patient contact are subject to ethics committee review and approval.

### Data management and analysis

All data will be collected on paper case report forms and pseudonymised data will be entered onto a secure shared electronic database, REDCap, hosted centrally at QMUL. Local coordinators and the UK team will provide regular monitoring to ensure accurate data collection and entry. All patient-identifiable data will be stored separately at the local research site in a locked and access-controlled filing system.

Data analysis will be discussed and agreed with the local research teams and formulated into a statistical analysis plan. The study’s international Advisory Group will be consulted if required. The local research team will take a leading role in the management and analysis of data. Data analysis will not be conducted until a statistical analysis plan has been agreed. Once signed off, the plans will be available on request.

The studies will first be analysed separately, where the mean outcome scores will be compared before and after the intervention. We will also combine the fully anonymised datasets from the three countries. This will make it possible to compare findings and experiences and to learn from commonalities and differences. The director of the Group will have access to the final complete dataset. Reasonable requests for the anonymised quantitative data, formalised through a data sharing agreement, will be possible one year after the study has concluded to allow for all primary publication activities to be completed.

#### Quantitative data analysis

The number of screened participants, eligible participants and of those who refused participation or were not approached will be recorded. The analysis will assess the number of intervention sessions received by patients and will collect data on drop-out (including reasons for drop-out if available) from treatment, which will inform the assessment of acceptability and feasibility of the interventions. The life domains and treatment aspects selected for discussion in the DIALOG+ sessions will be tabulated.

Descriptive statistics will be reported for socio-demographic data for all participants. To assess for any changes in outcomes for patients using DIALOG+, mean and standard deviations over two time points (baseline and after the intervention period) will be calculated, with significance tests.

#### Qualitative data analysis

Qualitative data will be analysed using qualitative content analysis [48] and will be conducted using NVivo qualitative analysis software. All interviews will be audio-recorded and transcribed verbatim for analysis. A researcher will remove all identifying information from the transcripts, including any references to patients, clinicians or local services.

A mixed deductive and inductive approach will be used to code the qualitative data. This will be based both on a coding framework developed alongside the interview topic guides to provide insights regarding the experiences of delivering and receiving the interviews, suggested adaptations to training material, barriers and facilitators of attendance and delivery, and the impact of DIALOG+ on routine meetings and care, as well as allowing for new codes to emerge inductively from the data.

## Dissemination

The researchers intend to publish the quantitative and qualitative findings from this study by October 2022. Dissemination will target different stakeholders, including mental health service commissioners and policymakers, clinicians, patients, carers, academics and the general public. This study is part of a research group which also aims to build sustainable research capacity. The dissemination plan therefore aims to inform research, policy and practice. The researchers plan to disseminate findings across Uganda, Bosnia and Herzegovina and Colombia, as well as regionally and globally. Dissemination will include peer-reviewed academic publications by researchers, conference presentations, and using platforms like Twitter and the Group and local research team websites.

## Discussion

People with chronic physical illness in primary care frequently experience comorbid psychological distress, and associated poor quality of life remains a key concern. However, there is limited evidence regarding the design and delivery of interventions for this population, especially in LMICs. DIALOG+ is used in existing routine patient-clinician meetings and is very low cost, and it may have the potential to help large numbers of patients with chronic physical conditions, mental distress and poor quality of life who are treated in primary care. This is particularly relevant in LMICs where resources for specialised services for such patients are scarce or non-existent. There is considerable evidence that DIALOG+ improves quality of life in patients with mental disorders, but also emerging evidence that it improves anxiety and depression and quality of life in patients with mental distress (Saleem S, Baig A, Sajun S, Bird VJ, Priebe S, Pasha A: A mixed methods exploration of structured patient-clinician communication with a solution focused approach (DIALOG+) in community treatment of patients with common mental health disorders in Pakistan, unpublished). This present study will build on this evidence base and assess the feasibility, experiences and outcomes of delivering the intervention in primary care settings in LMICs. Feasibility will be assessed by determining the extent to which the intervention is implemented as planned. Acceptability will be assessed through experiences as captured in interviews and focus groups with care providers and participants. Improvements from baseline to the end of the intervention period will be used to assess the impact of the intervention in this context. This trial will inform our understanding of the extent to which DIALOG+ needs to be flexibly adapted for new settings, or if the approach can be standardised across a range of patient groups and settings.

Task sharing and integrating mental health into primary care have been highlighted as crucial steps towards closing the mental health treatment gap. As a manualised, app-assisted intervention, DIALOG+ could be easily adapted for delivery by non-mental health professionals. This study will provide valuable insights into the potential for task-sharing and delivery in multiple settings. By conducting comparable trials in three health systems, including a mix of LMICs in three continents, our findings could have applicability across multiple settings.

Evidence from high-income settings suggests DIALOG+ is a cost-saving intervention for people with mental disorders. Research in high-income countries estimates that the top 5% of chronic patients utilise over 50% of all healthcare resources [49]. In low-resource settings, this intervention provides an opportunity to leverage existing routine meetings in primary care to deliver a cost-effective or cost-saving intervention. There is the potential to expand into other primary care settings or other meetings between care providers and patients with poor quality of life. We anticipate that anyone who has routine meetings with patients in primary care could potentially incorporate DIALOG+ into those meetings, providing structure and a way to improve patient quality of life and mental distress. We anticipate this would include, but not be limited to, doctors, nurses, counsellors, lay counsellors and associated community healthcare workers, and pharmacists. Process measures will help to better understand the staffing and resources required for wider implementation.

## Conclusion

The factors that lead to lowered quality of life are likely to be numerous and complex, and so a holistic intervention that concurrently addresses numerous aspects relating to patient quality of life, like DIALOG+, is likely to be more efficient and effective, rather than an intervention which focuses on specific aspects of their treatment or disease. The findings of this exploratory trial can inform the design of future full randomised controlled trials on DIALOG+ in primary care settings in LMICs.

## Data Availability

Anonymised data arising from the study will be available to external researchers upon reasonable request to the Principal Investigator (s.priebe@qmul.ac.uk) based on a signed data sharing agreement and only after the publication of the findings of the study by the research team. Study protocols are available on request.

## Abbreviations

BiH: Bosnia and Herzegovina
SARS-CoV-2 (COVID-19): Severe acute respiratory syndrome coronavirus 2
DSM: Diagnostic and Statistical Manual of Mental Disorders
EPS: Entidad Promotora de Salud, or Health Promoting Entity
GAD-7: Generalised Anxiety Disorder Assessment
GLOBE: The NIHR Global Health Research Group on Developing Psycho-social Interventions in Low- and Middle-Income Countries
HIV/AIDS: Human immunodeficiency virus/acquired immune deficiency syndrome
IPS: Institución Prestadora de Servicios, or Service Provider Institution
LMICs: Low- and middle-income countries
MANSA: Manchester Short Assessment of Quality of Life
MIAS: Modelo Integral de Atención en Salud, or comprehensive health care model
PAIS: Plan de Atención Integral en Salud, or comprehensive health care policy
PHQ-8/9: Patient Health Questionnaire
RIAS: Red Integral de Atención en Salud, comprehensive health care routes
SF-36/12: Physical Health Symptoms measure
SIX: Objective Social Outcomes Index
UBACC: University of California, San Diego Brief Assessment of Capacity to Consent
